# Cardioprotective Effects of Bosentan in Rats Subjected to Lung Ischemia–Reperfusion Injury

**DOI:** 10.3390/medicina61071298

**Published:** 2025-07-18

**Authors:** Şevki Mustafa Demiröz, Ayşegül Küçük, Esra Tekin, Sibel Söylemez, Hanife Yılmaz, Şaban Cem Sezen, Muharrem Atlı, Hüseyin Demirtaş, Abdullah Özer, Yusuf Ünal, Mustafa Arslan

**Affiliations:** 1 Department of Thoracic Surgery, Faculty of Medicine, Gazi University, Ankara 06510, Turkey; demirozsm@gmail.com; 2 Department of Physiology, Faculty of Medicine, Kutahya Health Sciences University, Kutahya 43000, Turkey; kucukaysegul@hotmail.com (A.K.); esra.tekin@ksbu.edu.tr (E.T.); 3 Application and Research Centre for Life Sciences, Gazi University, Ankara 06830, Turkey; soylemezsibel@gmail.com; 4 Department of Anesthesiology and Reanimation, Faculty of Medicine, Gazi University, Ankara 06510, Turkey; drhanifeyilmaz@outlook.com (H.Y.); yunal71@yahoo.com (Y.Ü.); 5 Department of Histology and Embryology, Kırıkkale University Faculty of Medicine, Kırıkkale 71450, Turkey; sezenscem@gmail.com (Ş.C.S.); muharrematli@gmail.com (M.A.); 6 Department of Cardiovascular Surgery, Faculty of Medicine, Gazi University, Ankara 06510, Turkey; drhuseyindemirtas@yahoo.com (H.D.); dr-abdozer@hotmail.com (A.Ö.); 7 Centre for Laboratory Animal Breeding and Experimental Research (GÜDAM), Gazi University, Ankara 06510, Turkey

**Keywords:** bosentan, lung ischemia–reperfusion, cardioprotection, oxidative stress, endothelin-1, myocardial injury, rat model

## Abstract

*Background and Objectives*: This study aimed to investigate the cardioprotective effects of bosentan, an endothelin receptor antagonist, in a rat model of lung ischemia–reperfusion (I/R) injury, with a focus on myocardial tissue involvement. *Materials and Methods*: Twenty-four male Wistar rats were randomly assigned to four groups: sham, bosentan, I/R, and I/R + bosentan. Lung I/R injury was induced by hilar clamping for 45 min, followed by 60 min of reperfusion. Bosentan (30 mg/kg) was administered intraperitoneally 30 min prior to the procedure. Myocardial tissue was evaluated histopathologically for structural disorganization, inflammation, fibrosis, and edema. TGF-β1 protein levels in myocardial tissue were compared across the groups using β-actin as the loading control. ELISA was used to quantify ET-1, NF-κB, and p53 levels, while spectrophotometric analysis was employed to assess MDA levels and the activities of SOD and CAT enzymes in heart tissue. *Results*: The I/R group exhibited significant myocardial disorganization, inflammation, and interstitial edema compared to the sham and bosentan groups. Bosentan treatment markedly ameliorated these histopathological alterations. Additionally, the I/R group showed elevated levels of ET-1, NF-κB, p53, and MDA, along with reduced SOD and CAT activities; these changes were significantly attenuated by bosentan administration. Bosentan treatment significantly reduced myocardial ET-1 levels (from 136.88 ± 5.02 to 120.18 ± 2.67 nmol/g, *p* = 0.003), NF-κB levels (from 0.87 ± 0.04 to 0.51 ± 0.03 ng/mg, *p* = 0.002), and TGF-β1 expression (from 1.72 ± 0.10 to 0.91 ± 0.08 relative units, *p* = 0.001). Moreover, bosentan increased antioxidant enzyme activities, elevating SOD levels from 21.45 ± 1.23 to 32.67 ± 1.45 U/mg protein (*p* = 0.001) and CAT levels from 15.22 ± 0.98 to 25.36 ± 1.12 U/mg protein (*p* = 0.002). *Conclusions*: Bosentan exerts cardioprotective effects in rats subjected to lung I/R injury by reducing myocardial damage, inflammation, and oxidative stress. These findings suggest that bosentan may serve as a potential therapeutic agent for preventing remote organ injury associated with pulmonary I/R.

## 1. Introduction

Bosentan is the first orally administered endothelin (ET) receptor antagonist approved for the treatment of pulmonary arterial hypertension (PAH) [1,2]. It provides significant clinical benefits by improving exercise capacity, alleviating symptoms, enhancing functional status, and slowing disease progression [2]. ET-1, the principal isoform of the endothelin family, plays a central role in vascular responses to hypoxia, ischemia, and other stimuli [1]. Its effects are mediated through ETA and ETB receptors, contributing to vasoconstriction, inflammation, and fibrosis in various tissues, including the heart [1,2]. Elevated ET-1 levels have been reported in patients with PAH and scleroderma [3]. Bosentan has shown benefits in systemic sclerosis-related complications [3,4].

Findings from experimental studies suggest broader applications beyond the current clinical indications [5,6,7,8,9]. Demirtaş et al. [10] demonstrated that bosentan reduces lower limb ischemia–reperfusion injury. Gong et al. [11] reported that bosentan modulates spinal cord ischemia–reperfusion injury via the VEGF pathway. Kazımoğlu et al. [12] showed that bosentan confers renal protection in the setting of ischemia–reperfusion injury. Gupta et al. [13] revealed that bosentan ameliorates myocardial ischemia–reperfusion injury. Okada et al. [14] highlighted the critical role of ET-1 in lung ischemia–reperfusion injury, suggesting that bosentan may offer therapeutic benefit in this context. The evaluation of myocardial injury markers is essential in assessing the extent of ischemia–reperfusion injury. ET-1 levels rise significantly during the early phase of myocardial ischemia–reperfusion injury [15]. Additionally, the Toll-like receptor 4 (TLR4)/nuclear factor kappa B (NF-κB) axis plays a crucial role in mediating inflammatory responses [16]. The quantification of β-actin is frequently used as a loading control in Western blot analyses for assessing myocardial injury [17]. Moreover, p53 expression is strongly associated with myocardial ischemia–reperfusion injury and may provide additional insight into the severity of tissue damage [18].

Bosentan may cause adverse effects such as headache, flushing, peripheral edema, and hepatotoxicity, warranting caution in clinical use [1,19,20]. This study was designed to investigate the effects of lung ischemia–reperfusion injury. Subsequently, myocardial tissue was analyzed to explore the potential systemic protective effects of bosentan. Through this work, we aim to contribute meaningful insights to the current literature.

Pulmonary I/R injury is clinically significant in conditions such as lung transplantation, pulmonary embolism, and major thoracic surgeries, where the transient interruption of pulmonary blood flow is common. This not only results in primary lung injury but may also trigger remote myocardial injury through systemic inflammatory responses and oxidative stress. Understanding protective strategies against such remote cardiac injury is crucial for improving perioperative outcomes and reducing morbidity and mortality in these settings [21].

## 2. Materials and Methods

This experimental study was conducted at the Gazi University Laboratory Animal Breeding and Experimental Research Center (GÜDAM) in accordance with the ARRIVE guidelines. The study protocol was approved by the Gazi University Animal Experiments Local Ethics Committee (G.Ü.ET-24.118), Ankara, Turkey. All the procedures complied with the National Institutes of Health Guidelines for the Care and Use of Laboratory Animals. In addition, all the researchers performing the animal procedures held valid laboratory animal experimentation certificates.

**Figure 1:**
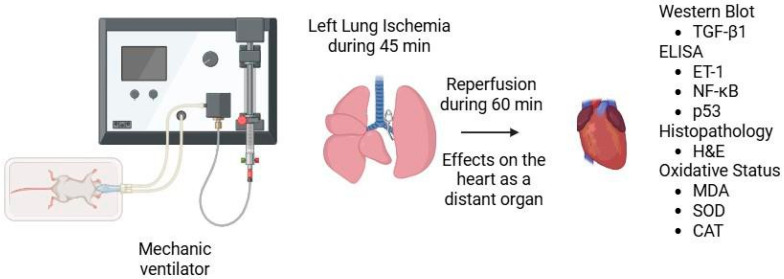
Graphical representation of the experimental method.

A total of 28 male Wistar rats (weighing 200–250 g) were obtained from GÜDAM and randomly divided into four groups (Figure 1). The animals were housed in clean cages with free access to standard chow and water under ad libitum conditions.

All the surgical procedures were performed under ketamine (50 mg/kg)-xylazine (5 mg/kg) anesthesia. A tracheostomy was performed, followed by endotracheal intubation. The rats were mechanically ventilated using an animal-specific ventilator (tidal volume: 0.1–0.2 mL/kg; respiratory rate: 30–50 breaths/min). Anesthesia was maintained with 2% inhaled isoflurane. To maintain euvolemia, 1 mL of intraperitoneal saline was administered every 30 min during the procedure. All the rats were heparinized with sodium heparin (500 IU/kg intraperitoneally). In the right lateral decubitus position, the left hemithorax was shaved and sterilized with a povidone–iodine solution. A thoracotomy was performed through the 5th intercostal space. The left hilum was visualized and carefully dissected. The total ischemia model was used; whole hilar structures (both pulmonary artery, vein, and bronchus) were clamped by the use of the YASARGIL^®^ Aneurysm Clip System (Aesculap, Tuttlingen, Germany).

Group I (Sham Group): A thoracotomy was then performed through the left fifth intercostal space, and the pulmonary hilum was exposed without clamping. After 105 min of ventilation, euthanasia was performed via intracardiac blood collection, and the heart tissue was harvested.

Group II (Bosentan Group): The rats received an intraperitoneal injection of bosentan (30 mg/kg in 1 mL saline) 30 min before the procedure. The same surgical steps described for the Control group were followed.

Group III (Ischemia–Reperfusion Group): A thoracotomy was performed through the 5th intercostal space. The left hilum is seen and dissected carefully. The total ischemia model was used; whole hilar structures (both pulmonary artery, vein, and bronchus) were clamped by the use of the YASARGIL^®^ Aneurysm Clip System for 45 min. The clamp was then removed to allow reperfusion for an additional 60 min. The 60 min reperfusion period was chosen based on prior studies demonstrating that significant biochemical and histopathological alterations occur within this time frame [10,11,12,13]. At the end of the procedure, euthanasia was carried out, and tissue samples were collected.

Group IV (Bosentan + Ischemia–Reperfusion Group): Rats received bosentan (30 mg/kg, i.p.) 30 min prior to surgery. Lung I/R was induced as described in Group III.

At the end of the period, the rats were sacrificed. After the application of 100 mg/kg of ketamine, intracardiac blood was taken, and the sacrificial process was performed, and heart tissue was harvested. Tissues intended for histological examination were fixed in 10% neutral-buffered formalin. After 72 h of fixation, the samples underwent routine processing, and paraffin blocks were prepared. Sections with a thickness of 4–5 µm were obtained from the paraffin blocks.

### 2.1. Histopathologic Analysis

Tissues fixed for 72 h in 10% neutral-buffered formalin were subjected to routine histological processing, and paraffin blocks were prepared. Sections with a thickness of 4–5 µm were obtained and stained with hematoxylin and eosin (H&E). The degree of inflammation was evaluated and graded as mild, moderate, or severe based on previously described criteria in the literature [21,22,23,24,25]. Histopathological differences between groups were recorded and statistically analyzed. Myocardial disorganization was defined as the disruption of the normal parallel arrangement of myocardial fibers, loss of cellular architecture, and irregular intercellular spaces. All the histopathological analyses were performed by investigators blinded to the experimental groups to minimize assessment bias.

Western blotting is a technique that enables the detection and quantification of specific proteins in tissue samples [26]. In this study, the TGF-β1 protein levels in myocardial tissue were compared across the groups, using β-actin as the loading control. Densitometric analysis of Western blot bands was performed using the ImageJ (Version 1.54g) software (NIH, Bethesda, MD, USA). The relative expression of TGF-β1 was calculated as the ratio of its band intensity to that of β-actin, which served as the loading control.

Initially, 30 mg of heart tissue was weighed using a precision scale. A volume of 500 µL of RIPA lysis buffer was added, and the samples were homogenized. Homogenates were centrifuged at 12,000 rpm for 15 min, and the supernatants were transferred to clean Eppendorf tubes and stored at –80 °C. Protein concentrations in the supernatants were determined using a BCA total protein assay kit. Based on these concentrations, the appropriate sample volume required to load 25 µg of protein was calculated.

For SDS-PAGE, a 10% resolving gel and a 4% stacking gel were prepared using the BIO-RAD Mini-PROTEAN system (Hercules, CA, USA), including gel casting stands, glass plates, and combs. After preparing the loading buffer, protein samples were loaded onto the gel. Electrophoresis was performed to separate proteins, which were subsequently transferred onto PVDF membranes. The membranes were blocked and incubated with primary and secondary antibodies, followed by chemiluminescence imaging to detect protein bands.

Heart tissues obtained after the I/R procedure were homogenized in nine volumes of cold phosphate-buffered saline (PBS). Following centrifugation at 4500 rpm for 15 min at 4 °C, the supernatants were collected. Levels of NF-κB, ET-1, and p53 in myocardial tissue were determined using enzyme-linked immunosorbent assay (ELISA). According to standard ELISA protocols, samples were incubated, washed, and then exposed to the substrate. Upon color development (blue), a stop solution was added to produce a final color change (yellow). Absorbance was measured spectrophotometrically, and concentrations were calculated based on standard curves generated using known concentrations.

Commercially available ELISA kits were used for the analysis (NF-κB: E-EL-R0674, Elabscience, Houston, TX, USA; ET-1: E-EL-R1458, Elabscience, Houston, TX, USA; p53: E0071Ra, Elabscience, Houston, TX, USA), and procedures were carried out in accordance with the manufacturer’s instructions.

### 2.2. Biochemical Analysis

Biochemical assays were performed on the myocardial tissue samples to evaluate oxidative stress levels and antioxidant enzyme activities. The heart tissues were homogenized in nine volumes of cold PBS and centrifuged at 4500 rpm for 15 min at 4 °C. The resulting supernatants were used for spectrophotometric measurements.

Malondialdehyde (MDA) levels, reflecting lipid peroxidation, were determined using the thiobarbituric acid reactive substances (TBARS) method. The results were expressed in nanomoles per gram of protein (nmol/g protein). Superoxide dismutase (SOD) activity was measured based on its ability to inhibit pyrogallol autoxidation, and values were expressed as international units per milligram of protein (IU/mg protein). Catalase (CAT) activity was assessed by monitoring the decomposition rate of hydrogen peroxide at 240 nm and expressed as IU/mg protein. Protein concentrations were measured using the Bradford assay to normalize enzyme activities. All the analyses were performed in triplicate, and average values were included in the statistical evaluation. The applied procedures align with the established methods used in ischemia–reperfusion models for assessing oxidative stress and antioxidant status [27,28,29,30,31,32,33].

### 2.3. Statistical Analysis

Statistical analyses were performed using IBM SPSS Statistics for Windows, version 26.0 (IBM Corp., Armonk, NY, USA). All numerical data were presented as mean ± standard error of the mean (SEM). Normality of data distribution was assessed using the Shapiro–Wilk test. One-way analysis of variance (ANOVA) was used to compare means among the four groups (sham, bosentan, I/R, and I/R + bosentan). When a significant difference was detected by ANOVA, post hoc pairwise comparisons were performed using the Tukey HSD test to determine differences between specific groups. A *p*-value of less than 0.05 was considered statistically significant. Statistical analysis was applied to both histopathological scoring parameters (e.g., myocardial disorganization, inflammation, interstitial fibrosis, edema, and cell swelling) and biochemical markers (ET-1, NF-κB, p53, MDA, SOD, and CAT).

Although no formal power calculation was performed, the group sizes (*n* = 6–8) were determined based on previous experimental studies, demonstrating that such sample sizes are sufficient to detect significant differences in biochemical and histopathological outcomes in rodent I/R models [23,33].

## 3. Results

### 3.1. Histopathologic Findings

**Figure 2:**
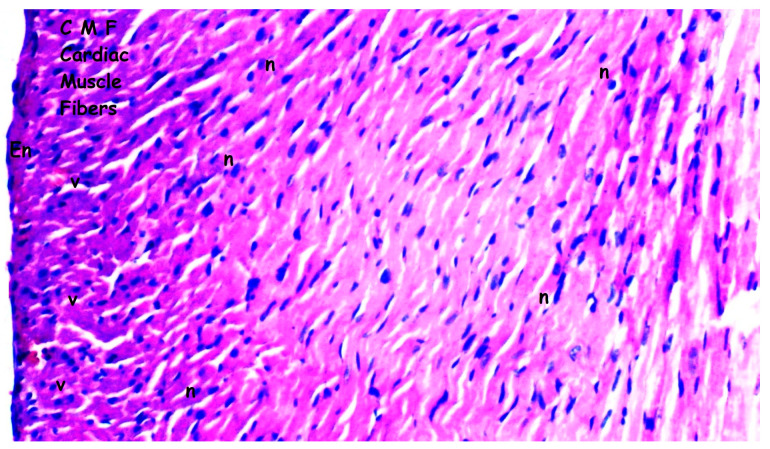
Representative images of histopathologic analysis obtained from hematoxylin and eosin staining. Sham group; CMF: cardiac muscle fibers; n: nucleus; v: ventricle; En: endothelium. Histological appearance of myocardial tissue in the sham group showing normal myocardial architecture (H&E staining, ×200 magnification).

**Figure 3:**
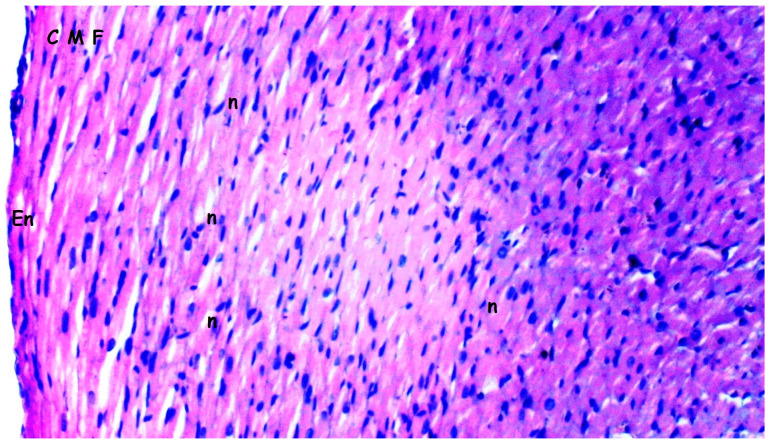
Representative images of histopathologic analysis obtained from hematoxylin and eosin staining. Bosentan group; CMF: cardiac muscle fibers; En: endothelium; n: nucleus n: nucleus; Histological appearance of myocardial tissue in the I/R group showing interstitial edema and inflammatory cell infiltration (H&E staining, ×200 magnification).

**Figure 4:**
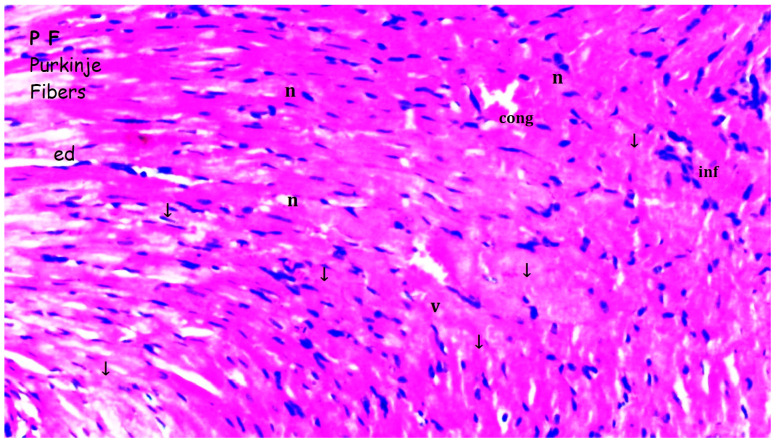
Representative images of histopathologic analysis obtained from hematoxylin and eosin staining. I/R group; PL: Purkinje fibers; n: nucleus; v: ventricle; conj: congestion; ↓: interstitial fibrosis; ed: interstitial edema; inf: inflammation.

**Figure 5:**
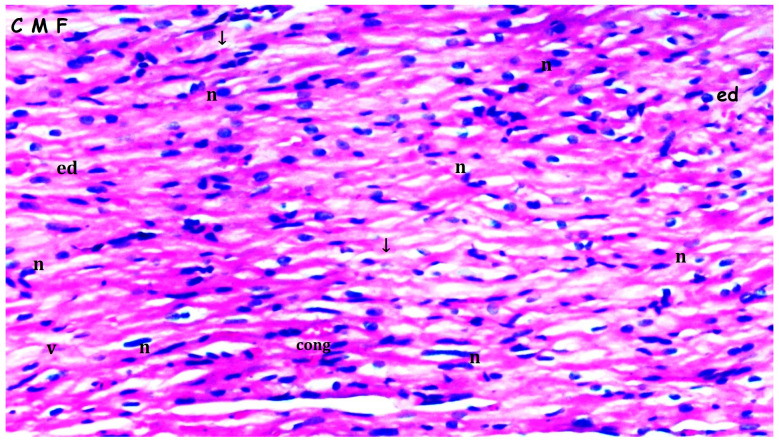
Representative images of histopathologic analysis obtained from hematoxylin and eosin staining. Bosentan + I/R; CMF: cardiac muscle fibers; n: nucleus; v: ventricle; ↓: interstitial fibrosis; ed: interstitial edema; conj: congestion.

Myocardial disorganization differed significantly between the groups (*p* = 0.028). It was observed more frequently in the I/R group compared to groups sham and bosentan (*p* = 0.013 for both). The B + IR group exhibited significantly less myocardial disorganization than the I/R group (*p* = 0.023) (Table 1, Figure 2, Figure 3, Figure 4 and Figure 5).

Inflammation and neutrophil infiltration also differed significantly among the groups (*p* = 0.012). These findings were significantly more pronounced in the I/R group compared to groups sham and bosentan (*p* = 0.003 and *p* = 0.012, respectively). The B + IR group showed a significant reduction in inflammation and neutrophil infiltration compared to the I/R group (*p* = 0.022) (Table 1, Figure 2, Figure 3, Figure 4 and Figure 5).

Interstitial fibrosis varied significantly between groups (*p* = 0.010), being more frequent in the I/R group compared to groups sham and bosentan (*p* = 0.003 and *p* = 0.010, respectively) (Table 1, Figure 2, Figure 3, Figure 4 and Figure 5).

Interstitial edema differed significantly among the groups (*p* = 0.010). It was observed more frequently in the I/R group than in groups sham and bosentan (*p* = 0.003 and *p* = 0.012, respectively), while the B + IR group showed significantly less edema than the I/R group (*p* = 0.022) (Table 1, Figure 2, Figure 3, Figure 4 and Figure 5).

The swelling of myocardial cells also differed significantly between groups (*p* = 0.013). It was more frequently observed in the I/R group compared to the groups sham and bosentan (*p* = 0.005 for both). The B + IR group showed significantly less swelling compared to the I/R group (*p* = 0.046) (Table 1, Figure 2, Figure 3, Figure 4 and Figure 5).

Myocardial necrosis was found to be similar across all the groups, with no statistically significant difference (*p* = 0.126) (Table 1, Figure 2, Figure 3, Figure 4 and Figure 5).

**Figure 6:**
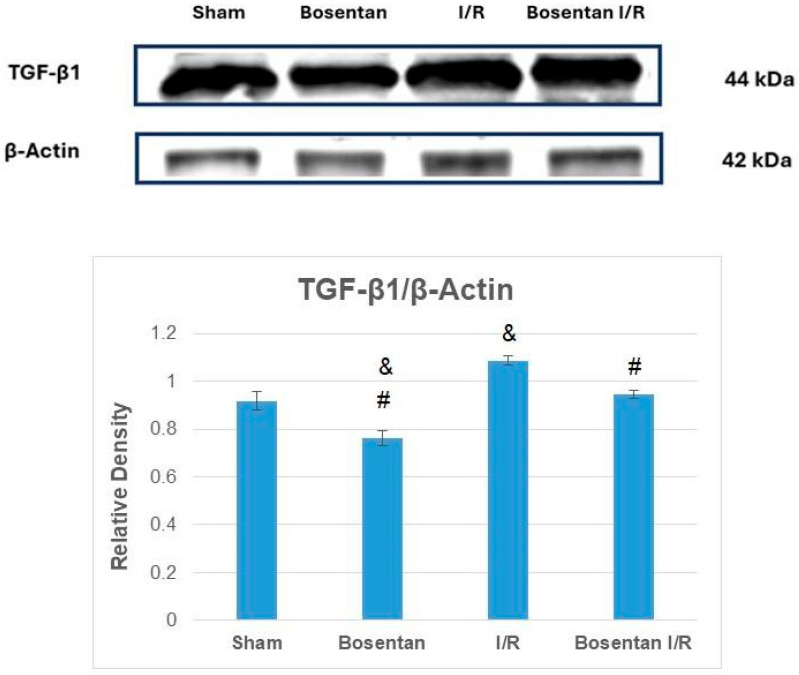
Representative images and graphs obtained from Western blot. & *p* < 0.05 compared to the sham group; # *p* < 0.05 compared to the I/R group.

TGF-β1 levels were significantly lower in the bosentan group compared to the sham group (*p* < 0.001). The highest TGF-β1 levels were observed in the I/R group, which were significantly higher than those in the sham, bosentan, and B + IR groups (*p* < 0.001, *p* < 0.001, and *p* = 0.001, respectively). The bosentan I/R group had similar TGF-β1 levels to the sham group (Figure 6).

**Figure 7:**
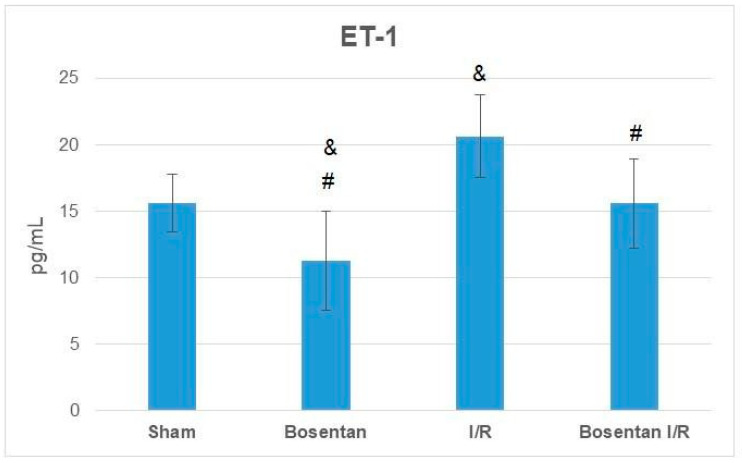
ET-1 levels obtained from ELISA. & *p* < 0.05 compared to the sham group; # *p* < 0.05 compared to the I/R group.

ET-1 levels were significantly lower in the bosentan group compared to the sham group (*p* = 0.015). The highest ET-1 levels were observed in the I/R group, which were significantly higher than those in the sham, bosentan, and B + IR groups (*p* = 0.005, *p* = 0.001, and *p* = 0.005, respectively). ET-1 levels in the B + IR group were comparable to those in the sham group (Figure 7).

**Figure 8:**
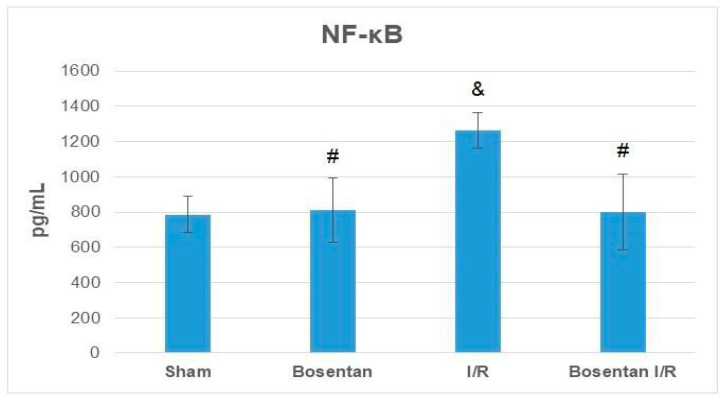
NF-κB levels obtained from ELISA. & *p* < 0.05 compared to the sham group; # *p* < 0.05 compared to the I/R group.

NF-κB levels in the bosentan group were similar to those in the sham group (*p* > 0.05). The highest levels were recorded in the I/R group, which were significantly elevated compared to the sham, bosentan, and B + IR groups (*p* = 0.001, *p* = 0.001, and *p* = 0.002, respectively). NF-κB levels in the B + IR group were similar to those in the sham group (Figure 8).

**Figure 9:**
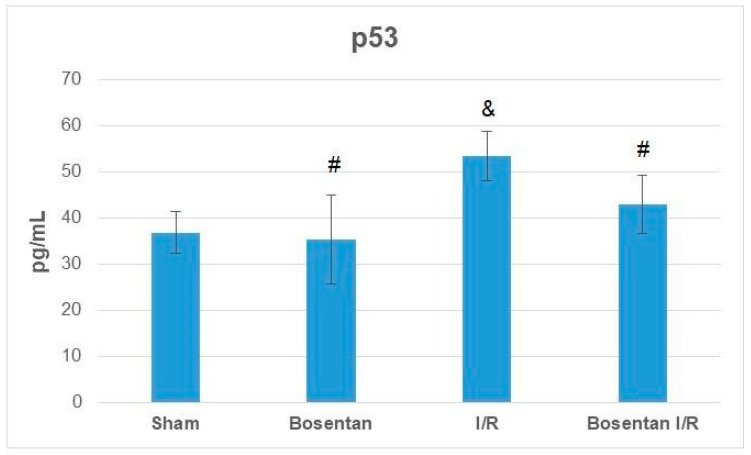
p53 levels obtained from ELISA. & *p* < 0.05 compared to the sham group; # *p* < 0.05 compared to the I/R group.

p53 protein levels in the bosentan group were also comparable to those in the sham group (*p* > 0.05). The I/R group demonstrated the highest levels, which were significantly higher than those in the sham, bosentan, and B + IR groups (*p* = 0.001, *p* = 0.001, and *p* = 0.007, respectively). p53 levels in the B + IR group were similar to those in the sham group (Figure 9).

### 3.2. Biochemical Findings

A significant difference in malondialdehyde (MDA) levels in heart tissue was observed among the groups (*p* < 0.001). MDA levels were significantly higher in the I/R group compared to groups sham and bosentan (*p* < 0.001 for both). The B + IR group showed significantly lower MDA levels than the I/R group (*p* = 0.003). (Table 2)

A significant difference was also observed in superoxide dismutase (SOD) enzyme activity among the groups (*p* < 0.001). SOD activity was significantly lower in the I/R group compared to groups sham and bosentan (*p* < 0.001 for both). In contrast, the B + IR group demonstrated significantly higher SOD activity than the I/R group (*p* < 0.001). (Table 2)

Similarly, catalase (CAT) enzyme activity differed significantly among the groups (*p* < 0.001). CAT activity was significantly lower in the I/R group compared to groups sham and bosentan (*p* < 0.001 and *p* = 0.002, respectively). CAT activity in the B + IR group was significantly higher than in the I/R group (*p* = 0.008). (Table 2)

## 4. Discussion

In this study, we demonstrated that bosentan exerts significant cardioprotective effects against lung ischemia–reperfusion injury in rats, evidenced by reduced myocardial inflammation, oxidative stress, and fibrosis. Ischemia–reperfusion injury (IRI) of the lung represents a rapid and multifactorial pathological process that paradoxically results in further tissue damage during the restoration of blood flow, despite its necessity to prevent irreversible injury [34]. Key pathophysiological components include epithelial cell damage, cytokine release, damage-associated molecular patterns (DAMPs), and the activation of alveolar macrophages, natural killer cells, and neutrophils [34]. Reactive oxygen species (ROS) production subsequently triggers lipid peroxidation, membrane injury, and amplification of inflammation [34]. Moreover, increased levels of free radicals have been documented in the systemic circulation following lung IRI, suggesting remote organ involvement, including the myocardium [35]. Elevated malondialdehyde (MDA) levels observed in the I/R group in our study are consistent with these findings and reflect oxidative damage. Importantly, the bosentan treatment significantly reduced MDA levels in the I/R + Bosentan group, while also enhancing antioxidant enzyme activities such as SOD and CAT, indicating that bosentan mitigates oxidative stress in myocardial tissue.

Oxidative stress not only damages cellular membranes but also disrupts DNA integrity, often through the overactivation of poly-ADP-ribose polymerase (PARP) [34]. Toll-like receptors (TLRs), particularly TLR4, are critical mediators in the progression of lung IRI. TLR4 recognizes endogenous DAMPs such as high mobility group box 1 (HMGB1), oxidized phospholipids, fibronectin, and heat shock proteins, thereby initiating inflammatory signaling via pathways like TLR4/TRIF [34,36]. Our findings of significantly elevated NF-κB levels in the I/R group, which were attenuated by bosentan, support the hypothesis that endothelin receptor blockade dampens TLR4-mediated NF-κB activation, contributing to its anti-inflammatory effects.

Multiple intracellular signaling pathways—including HGF/c-Met, Wnt, BMP, Hippo-YAP, Notch, PI3K/Akt, and TGF-β—have been implicated in IRI pathogenesis, particularly in modulating apoptosis and fibrosis [36]. Among these, TGF-β plays a dual role: promoting tissue remodeling and repair, while also contributing to fibrosis when excessively activated. TGF-β1 in particular promotes fibroblast proliferation and differentiation into myofibroblasts, a hallmark of myocardial fibrosis [36,37]. In our study, myocardial TGF-β1 levels were significantly increased in the I/R group but normalized with bosentan treatment, suggesting that bosentan may protect the heart by modulating fibrogenic signaling pathways in addition to inflammation and oxidative stress. The interstitial fibrosis observed in our study likely represents early extracellular matrix changes rather than fully developed mature fibrosis, as significant collagen deposition usually requires longer periods post-injury. The absence of significant myocardial necrosis in our study may be due to the relatively short ischemia duration, which primarily induced reversible myocardial injury rather than irreversible cell death.

Following an ischemic event, reperfusion introduces a hyperoxic environment, which exacerbates tissue injury through excessive ROS production [38]. Cardiac fibroblasts demonstrate greater resistance to oxygen toxicity than other cardiac cells [39]. As a result, fibroblasts become the predominant cell type within the infarcted region [39]. Hyperoxia promotes the differentiation of cardiac fibroblasts into myofibroblasts, a process largely mediated by TGF-β [39]. However, TGF-β also exerts cardiovascular protective effects, including the reduction in atherosclerosis, the enhancement in carotid plaque stability, and protection against stroke [40].

In addition to its myocardial effects, the behavior of TGF-β in pulmonary tissue following I/R injury further supports its dual role in injury and repair processes. TGF-β levels have been reported to increase in the lungs following intestinal ischemia–reperfusion (I/R) injury [41]. This observation suggests that TGF-β may contribute positively to lung repair processes [41]. In another study, TGF-β was shown to facilitate the healing of ischemia–reperfusion injury by upregulating BCL-2 expression and downregulating TNF-α levels [42]. Moreover, TGF-β demonstrated beneficial effects in lung reperfusion injury after prolonged ischemic periods [43].

The NF-κB signaling pathway is another critical mediator of I/R injury, playing a pivotal role in the regulation of apoptosis, inflammation, oxidative stress, and fibrosis [36]. Apoptosis is a major contributor to I/R injury [16]. The activation of TLR4 during I/R triggers the NF-κB pathway, leading to increased cytokine production and the expression of genes related to inflammation and apoptosis [16]. Consequently, I/R promotes both apoptosis and autophagy via the TLR4/NF-κB pathway through the regulation of various genes [16]. Additionally, p53 is activated during myocardial I/R, where it contributes to the induction of apoptosis, necroptosis, autophagy, ferroptosis, and oxidative stress [18].

TGF-β1 activation is also a key mechanism in the development of lung fibrosis. In various models, inhibition of TGF-β1 signaling has been associated with attenuation of pulmonary fibrosis. In parallel, potent vasoconstrictors such as endothelins accumulate during lung I/R injury and exacerbate tissue damage. Both the lungs and heart can be adversely affected by I/R injury. Bosentan, an endothelin receptor antagonist, may exert protective effects in lung I/R injury and associated distant organ damage by modulating TGF-β1 expression.

In our study, TGF-β1 protein levels were elevated in heart tissue in the I/R group. However, bosentan treatment significantly reduced TGF-β1 expression. In the Bosentan + I/R group, TGF-β1 levels were decreased compared to the I/R group and became comparable to those in the sham group. Recent studies have highlighted the central role of TGF-β1 and NF-κB signaling pathways in mediating inflammation and fibrosis during myocardial I/R injury, offering potential therapeutic targets for mitigating cardiac damage [36]. The timing and dose of bosentan administration in our study were selected based on prior experimental studies demonstrating significant protective effects when administered 15–60 min before ischemic injury [10,11,12,13].

While primarily indicated for pulmonary arterial hypertension, bosentan may also hold promise for reducing remote myocardial injury associated with pulmonary ischemia–reperfusion events, such as those occurring during lung transplantation or thoracic surgery. However, the translation of these findings to clinical practice requires further human studies.

In conclusion, bosentan demonstrated anti-inflammatory and anti-apoptotic effects by reducing endothelin-1, NF-κB, and p53 levels in myocardial tissue. Additionally, bosentan exhibited therapeutic potential in mitigating distant organ (cardiac) injury secondary to lung I/R, likely through the suppression of TGF-β1 signaling. These findings are consistent with earlier studies on bosentan’s protective effects in renal, spinal cord, and myocardial I/R models [10,11,12,13] and expand our understanding by demonstrating its efficacy in preventing remote myocardial damage in pulmonary I/R.

### 4.1. Study Limitations

This study has certain limitations. First, the sample size is relatively small, which may limit the generalizability of our findings. Second, the experimental model utilized only male rats; thus, potential sex-related differences in response to bosentan remain unexplored. Third, the study focused on short-term outcomes; longer-term investigations are warranted to determine whether bosentan provides sustained protection against fibrosis and functional decline. Additionally, while the biochemical and histological parameters assessed are informative, future studies incorporating molecular markers of apoptosis and fibrosis (e.g., BCL-2, caspases, and α-SMA) could provide deeper mechanistic insight. A further limitation of our study is the absence of functional cardiac assessments, such as echocardiography or invasive hemodynamic measurements, which could provide valuable insights into the clinical implications of our biochemical and histological findings. A further limitation of our study is the absence of electrocardiographic monitoring or other functional assessments to detect potential complications, such as arrhythmias, which may occur during ischemia–reperfusion injury. Additionally, the relatively small sample size may limit the generalizability of our findings. Additionally, serum cardiac troponin levels were not measured, which could have provided additional functional evidence of myocardial injury.

Additionally, nitric oxide levels or its metabolites were not measured in this study due to technical and financial constraints, although such assessments could offer further mechanistic understanding of the interplay between endothelin and NO pathways in ischemia–reperfusion injury.

### 4.2. Clinical Implications and Future Directions

While our study is preclinical in nature, the findings suggest potential translational relevance. If confirmed in future human studies, bosentan may serve as a therapeutic option for attenuating cardiac complications secondary to pulmonary ischemia–reperfusion injury, especially in settings such as lung transplantation, pulmonary embolism, or major thoracic surgery. This approach could help limit remote organ damage, reduce perioperative morbidity, and improve patient outcomes.

## 5. Conclusions

In conclusion, this study demonstrates that bosentan exerts significant cardioprotective effects in a rat model of lung IRI. Bosentan administration resulted in markedly reduced myocardial disorganization, inflammation, and interstitial edema, alongside improved histopathological integrity. Biochemical analysis further revealed decreased levels of ET-1, nuclear factor kappa B (NF-κB), and p53, accompanied by reduced MDA concentrations and enhanced antioxidant enzyme activities, specifically SOD and CAT. These findings indicate that bosentan mitigates oxidative stress, inflammation, and apoptosis in myocardial tissue exposed to pulmonary I/R insult. Notably, the attenuation of transforming growth factor beta 1 (TGF-β1) expression suggests an additional antifibrotic effect, potentially limiting long-term myocardial remodeling. Collectively, these results highlight bosentan’s potential as a therapeutic agent not only in pulmonary arterial hypertension but also in the prevention of remote cardiac injury following lung ischemia–reperfusion events.

## Figures and Tables

**Table 1:** Histopathological findings of heart tissue [Mean ± SEM]. Histopathological findings of heart tissue [Mean ± SEM] (sham, *n* = 6; bosentan, *n* = 6; I/R, *n* = 8; bosentan + I/R, *n* = 8).

	Group Sham (*n* = 6)	Group Bosentan (*n* = 6)	Group I/R (*n* = 8)	Group Bosentan + I/R (*n* = 8)	*p* Value (ANOVA)
[23,33] Myocardial disorganization	0.50 ± 0.22	0.50 ± 0.22	1.25 ± 0.16 *,&	0.63 ± 0.18+	0.028
Inflammation and neutrophil infiltration	0.33 ± 0.21	0.50 ± 0.22	1.25 ± 0.16 *,&	0.63 ± 0.18+	0.012
Interstitial fibrosis	0.33 ± 0.21	0.50 ± 0.22	1.25 ± 0.16 *,&	0.75 ± 0.16	0.010
Interstitial edema	0.33 ± 0.21	0.50 ± 0.22	1.25 ± 0.16 *,&	0.63 ± 0.18 +	0.012
Swelling of myocardial cells	0.33 ± 0.21	0.33 ± 0.21	1.13 ± 0.13 *,&	0.63 ± 0.18 +	0.013
Myocardial necrosis	0.17 ± 0.17	0.33 ± 0.21	0.75 ± 0.16	0.63 ± 0.18	0.126

* *p* < 0.05: compared to group sham; & *p* < 0.05: compared to group bosentan; + *p* < 0.05: compared to group I/R.

**Table 2:** Heart tissue oxidant status findings [Mean ± SEM]. Heart tissue oxidant status findings [Mean ± SEM] (sham, *n* = 6; bosentan, *n* = 6; I/R, *n* = 8; bosentan + I/R, *n* = 8).

	Group Sham (*n* = 6)	Group Bosentan (*n* = 6)	Group I/R (*n* = 8)	Group Bosentan + I/R (*n* = 8)	*p* Value (ANOVA)
MDA (nmol/g protein)	111.22 ± 2.94	112.39 ± 3.41	136.88 ± 5.02 *,&	120.18 ± 2.67 +	<0.001
SOD (IU/mg protein)	34.18 ± 2.35	28.73 ± 2.85	18.22 ± 1.66 *,&	26.17 ± 1.73 +	<0.001
CAT (IU/mg protein)	52.21 ± 2.36	51.24 ± 1.17	38.16 ± 1.59 *,&	50.40 ± 2.32+	<0.001

* *p* < 0.05: compared to group sham; & *p* < 0.05: compared to group bosentan; + *p* < 0.05: compared to group I/R.

## Data Availability

The datasets used and/or analyzed during the current study are available from the corresponding author on reasonable request.

## References

[1] Cheng J.W.M. (2003). Bosentan: A dual endothelin receptor antagonist for the management of pulmonary hypertension. Heart Dis..

[2] Rubin L.J., Roux S. (2002). Bosentan: A dual endothelin receptor antagonist. Expert Opin. Investig. Drugs.

[3] Rubin L.J., Badesch D.B., Barst R.J., Galiè N., Black C.M., Keogh A., Pulido T., Frost A., Roux S., Leconte I. (2002). Bosentan therapy for pulmonary arterial hypertension. N. Engl. J. Med..

[4] Parisi S., Bruzzone M., Di Vittorio C.C., Laganà A., Peroni C., Fusaro E. (2014). Efficacy of bosentan in the treatment of Raynaud’s phenomenon in patients with systemic sclerosis never treated with prostanoids. Reumatismo.

[5] Ozturk L., Dogan H.T., Kilicarslan A., Aydin M.E., Ozer A., Demirtas H., Kilic Y., Iriz E., Kucuk A., Bayraktar A.C. (2017). Effect of different doses of pregabalin on skeletal muscle ischaemia-reperfusion injury in rat. Bratisl. Med. J..

[6] Özer A., Demirtaş H., Çomu F.M., Erer D., Kılıç Y., Mardin B., Arslan M., Küçük A., Oktar G.L. (2018). Protective effect of erdosteine on erythrocyte deformability in a rat model of lower limb ischemia/reperfusion injury. Turk. J. Med. Sci..

[7] Demirtas H., Özer A., Yıldırım A.K., Dursun A.D., Sezen Ş.C., Arslan M. (2025). Protective Effects of BPC157 on Liver, Kidney, and Lung Distant Organ Damagein Ratswith Experimental Lower-Extremity Ischemia–Reperfusion Injury. Medicina.

[8] Özer A., Comu F.M., Demirtas H., Kılıç Y., Mardin B., Öztürk L., Iriz E., Arslan M., Küçük A. (2017). Effect of different doses of pregabalin on erythrocyte deformability in rats with lower limb ischemia reperfusion injury. Anaesth. Pain Intensive Care.

[9] Özer A., Erel S., Küçük A., Demirtaş H., Sezen Ş.C., Boyunağa H., Oktar G.L., Arslan M. (2024). Evaluation of the effect of enriched hydrogen saline solution on distant organ (lung) damage in skeletal muscle ischemia reperfusion in rats. Sci. Prog..

[10] Demirtaş H., Özer A., Gülcan M.B., Yığman Z., Küçük A., Tekin E., Ünal Y., Dursun A., Dağlı A., Arslan M. (2025). Protective Effects of Bosentan via Endothelin Receptor Antagonism in Experimental Ischemia-Reperfusion Injury in the Lower Limb of Rats. Drug Des. Devel. Ther..

[11] Gong S., Seng Z., Wang W., Lv J., Dong Q., Yan B., Peng L., He X. (2015). Bosentan protects the spinal cord from ischemia reperfusion injury in rats through vascular endothelial growth factor receptors. Spinal Cord.

[12] Kazimoglu H., Uysal E., Dokur M., Gurer A.O., Batcioglu K., Uyumlu B.A., Petekkaya E., Karadag M. (2020). Comparison of the protective effects of selective endothelin-a receptor antagonist, ambrisentan, and dual endothelin-A/B receptor antagonist, bosentan, in experimental renal ischemia reperfusion injury. Bratisl. Lek. Listy.

[13] Gupta S.K., Saxena A., Singh U., Arya D.S. (2005). Bosentan, the mixed ETA-ETB endothelin receptor antagonist, attenuated oxidative stress after experimental myocardial ischemia and reperfusion. Mol. Cell Biochem..

[14] Okada M., Yamashita C., Okada M., Okada K. (1995). Contribution of endothelin-1 to warm ischemia/reperfusion injury of the rat lung. Am. J. Respir. Crit. Care Med..

[15] Tamareille S., Terwelp M., Amirian J., Felli P., Zhang X.Q., Barry W.H., Smalling R.W. (2013). Endothelin-1 Release during the Early Phase of Reperfusion Is a Mediator of Myocardial Reperfusion Injury. Cardiology.

[16] Zhao Z., Qu F., Liu R., Xia Y. (2020). Differential expression of miR-142-3p protects cardiomyocytes from myocardial ischemia-reperfusion via TLR4/NFkB axis. J. Cell Biochem..

[17] Zhang R., Yang D., Zhou C., Cheng K., Liu Z., Chen L., Fang L., Xie P. (2012). β-Actin as a loading control for plasma-based Western blot analysis of major depressive disorder patients. Anal. Biochem..

[18] Zhu X.Z., Qiu Z., Lei S.Q., Leng Y., Li W.Y., Xia Z.Y. (2025). The Role of P53 in Myocardial Ischemia-Reperfusion Injury. Cardiovasc. Drugs Ther..

[19] Chin K., Channick R. (2004). *Bosentan*. Expert Rev. Cardiovasc. Ther..

[20] Eriksson C., Gustavsson A., Kronvall T., Tysk C. (2011). Hepatotoxicity by bosentan in a patient with portopulmonary hypertension: A case-report and review of the literature. J. Gastrointestin Liver Dis..

[21] Hausenloy D.J., Yellon D.M. (2013). Myocardial ischemia-reperfusion injury: A neglected therapeutic target. J. Clin. Investig..

[22] Fiser S.M., Tribble C.G., Long S.M., Kaza A.K., Cope J.T., Laubach V.E., Kern J.A., Kron I.L. (2001). Lung transplant reperfusion injury involves pulmonary macrophages and circulating leukocytes in a biphasic response. J. Thorac. Cardiovasc. Surg..

[23] Demirtas H., Yildirim A.K., Ozer A., Dursun A.D., Sezen S.C., Kucuk A., Arslan M. (2025). Protective Effects of Metformin in Non-Diabetic Rats with Experimentally Induced Lower Extremity Ischemia–Reperfusion Injury. Turk. J. Vasc. Surg..

[24] Demirtas H., Ozer A., Yigit D., Dursun A.D., Kosa C., Kucuk A., Simsek E., Arslan M. (2025). In-Vivo Antioxidant and Therapeutic Effects of Ellagic Acid on Ischemia–Reperfusion Injury in Skeletal Muscle. Turk. J. Vasc. Surg..

[25] Iriz E., Iriz A., Take G., Ozgul H., Oktar L., Demirtas H., Helvacioglu F., Arslan M. (2015). Iloprost and vitamin C attenuates acute myocardial injury induced by suprarenal aortic ischemia-reperfusion in rabbits. Bratisl. Med. J..

[26] Mahmood T., Yang P.C. (2012). Western blot: Technique, theory, and trouble shooting. N. Am. J. Med. Sci..

[27] Durak I., Canbolat O., Kavutçu M., Oztürk H.S., Yurtarslani Z. (1996). Activities of total, cytoplasmic, and mitochondrial superoxide dismutase enzymes in sera and pleural fluids from patients with lung cancer. J. Clin. Lab. Anal..

[28] Paglia D.E., Valentine W.N. (1967). Studies on the quantitative and qualitative characterization of erythrocyte glutathione peroxidase. J. Lab. Clin. Med..

[29] Aebi H. (1974). Catalase. Methods of Enzymatic Analysis.

[30] Hashimoto S. (1974). A new spectrophotometric assay method of xanthine oxidase in crude tissue homogenate. Anal. Biochem..

[31] Kara H., Ozer A., Arpaci H., Demirtas H., Comu F.M., Oktar G.L., Erer D., Kucuk A., Arslan M. (2015). Effect of alprostadil on erythrocyte deformability in ischemia reperfusion injury. Bratisl. Med. J..

[32] Mihara M., Uchiyama M. (1978). Determination of malonaldehyde precursor in tissues by thiobarbituric acid test. Anal. Biochem..

[33] Demirtaş H., Yıldırım A.K., Özer A., Dursun A.D., Sezen Ş.C., Yığman Z., Küçük A., Arslan M. (2025). Potential protective effects of boldine in rat with an experimental myocardial ischemia-reperfusion model. J. Updates Cardiovasc. Med..

[34] Laubach V.E., Sharma A.K. (2016). Mechanisms of lung ischemia-reperfusion injury. Curr. Opin. Organ. Transplant..

[35] Gielis J.F., Boulet G.A., Briedé J.J., Horemans T., Debergh T., Kussé M., Cos P., Van Schil P.E. (2015). Longitudinal quantification of radical bursts during pulmonary ischaemia and reperfusion. Eur. J. Cardiothorac. Surg..

[36] Zhang M., Liu Q., Meng H., Duan H., Liu X., Wu J., Gao F., Wang S., Tan R., Yuan J. (2024). Ischemia-reperfusion injury: Molecular mechanisms and therapeutic targets. Signal Transduct. Target. Ther..

[37] Wu H.Y., Wu J.L., Ni Z.L. (2019). Overexpression of microRNA-202-3p protects against myocardial ischemia-reperfusion injury through activation of TGF-β1/Smads signaling pathway by targeting TRPM6. Cell Cycle.

[38] Li Y., Cai M., Sun Q., Liu Z., Cardounel A.J., Swartz H.M., He G. (2013). Hyperoxia and transforming growth factor β1 signaling in the post-ischemic mouse heart. Life Sci..

[39] Roy S., Khanna S., Azad A., Schnitt R., He G., Weigert C., Ichijo H., Sen C.K. (2010). Fra-2 mediates oxygen-sensitive induction of transforming growth factor beta in cardiac fibroblasts. Cardiovasc. Res..

[40] Redondo S., Santos-Gallego C.G., Tejerina T. (2007). TGF-beta1: A novel target for cardiovascular pharmacology. Cytokine Growth Factor Rev..

[41] Fu X.B., Yang Y.H., Sun T.Z., Gu X.-M., Jiang L.-X., Sun X.-Q., Sheng Z.-Y. (2000). Effect of intestinal ischemia-reperfusion on expressions of endogenous basic fibroblast growth factor and transforming growth factor betain lung and its relation with lung repair. World J. Gastroenterol..

[42] Grünenfelder J., Miniati D.N., Murata S., Falk V., Hoyt E.G., Robbins R.C. (2002). Up-regulation of Bcl-2 through hyperbaric pressure transfection of TGF-beta1 ameliorates ischemia-reperfusion injury in rat cardiac allografts. J. Heart Lung Transplant..

[43] Daddi N., Suda T., D’Ovidio F., Kanaan S.A., Tagawa T., Grapperhaus K., Kozower B.D., Ritter J.H., Yew N.S., Mohanakumar T. (2002). Recipient intramuscular cotransfection of naked plasmid transforming growth factor beta1 and interleukin 10 ameliorates lung graft ischemia-reperfusion injury. J. Thorac. Cardiovasc. Surg..

